# Cryptic and Non-Cryptic Diversity in Cleptoparasitic Bees of the Genus *Stelis* Panzer, 1806, Subgenus *Stelidomorpha* Morawitz, 1875, with a Description of New Species from the Arabian Peninsula (Hymenoptera, Megachilidae) †

**DOI:** 10.3390/insects16101030

**Published:** 2025-10-06

**Authors:** Max Kasparek, Christian Schmid-Egger, Huw Roberts

**Affiliations:** 1 Independent Researcher, Mönchhofstr. 16, 69120 Heidelberg, Germany; 2 Independent Researcher, Fischerstr. 1, 10317 Berlin, Germany; schmid-egger@gmx.de; 3 Independent Researcher, Al Ain, United Arab Emirates; hgbroberts@gmail.com

**Keywords:** hidden diversity, species delimitation, new species, putative species, cuckoo bees, Anthidiini, taxonomy, genetic barcoding, COI gene

## Abstract

**Simple Summary:**

Wild bees of the genus *Stelis* are cleptoparasitic, meaning they do not construct their own nests. Instead, they lay their eggs in the nests of other bee species, exploiting the provisions meant for the host’s offspring. Because of this behaviour, they are commonly referred to as cuckoo bees. In a revision of the subgenus *Stelidomorpha*, in which we applied both morphometric and genetic approaches, we identified two new species on the Arabian Peninsula and elevated one previously recognized subspecies to full species status. Additionally, we discovered that *Stelis nasuta* (Latreille, 1809) comprises three genetically distinct lineages with discrete geographic distributions. Statistical species delimitation analyses confirmed that these lineages show no overlap in the mitochondrial COI gene, which was used as a genetic marker. While these findings would normally justify their recognition as separate species, we adopted a conservative approach and did not formally describe new taxa due to the absence of discernible phenotypic differences. This study highlights the limited understanding of Palaearctic bee diversity and demonstrates that novel species can be uncovered both through expanded geographic sampling and the application of advanced methodological approaches.

**Abstract:**

Cleptoparasitic bees of the subgenus *Stelis* (*Stelidomorpha*) occur mainly in the Mediterranean and Middle East. In this study, we elevate *Stelis aegyptiaca* ssp. *canaria* Warncke, 1992 to species rank (*S. canaria* Warncke, 1992) and describe two new species, *Stelis alainensis* Kasparek sp. nov. and *Stelis surica* Kasparek sp. nov., both discovered in Oman and the United Arab Emirates. Morphological differences between these species and their closest relatives (*S. aegyptiaca* Radoszkowski, 1876, *S. pentelica* Mavromoustakis, 1963, and *S. nasuta* (Latreille, 1809)) are corroborated by genetic divergence in the mitochondrial COI barcode region, with Kimura 2-parameter (K2P) distances of 7.6–15.2%. A notable case is *Stelis nasuta*, which shows deep genetic subdivision into three clusters: (1) Iberian Peninsula and North Africa, (2) southeastern France, Italy, and the Balkans, (3) eastern Balkans, Turkey, and the Levant. Moderate genetic K2P distances of 2.9–3.3% complicated species delimitation. Analyses with ABGD, ASAP, bPTP, and RESL algorithms consistently supported recognition of these lineages as putative species. As multivariate analyses of 11 morphometric traits revealed no consistent diagnostic differences, we treat these lineages as phylospecies rather than formal taxa. Our findings demonstrate that bee diversity in the Palaearctic remains underestimated, and that expanded sampling and integrative approaches continue to reveal hidden lineages.

## 1. Introduction

The bee tribe Anthidiini includes a few genera whose members pursue a cleptoparasitic mode of life. The female of these species lays one or more eggs in a cell of the host species and in most cases the adult parasite then leaves, and the parasite larva feeds on the food that had been provided for the host larva [1]. Such bees are usually called “cuckoo bees”. In the Anthidiini, cleptoparasitism is documented in seven genera. The majority of species belong to the widely distributed genera *Stelis* and *Euaspis*, while a few species are associated with *Austrostelis* Michener and Griswold, 1994, *Afrostelis* Cockerell, 1931, *Hoplostelis* Dominique, 1898, *Larinostelis* Michener and Griswold, 1994, and *Xenostelis* Baker, 1999. Molecular data places these cleptoparasitic genera into two distinct groups [2]. The first, referred to as the *Stelis* group, includes the genera *Afrostelis*, *Euaspis*, *Larinostelis*, *Stelis*, and *Xenostelis*. The second, the *Anthodioctes* group, comprises *Hoplostelis* and *Austrostelis*.

In the Holarctic realm, by far the most cleptoparasitic anthidiine species have been attributed to the genus *Stelis*. A total of 98 species were recognized globally in 2007 [1], with a further ten species subsequently described from the Nearctic region [3]. In the Eastern Hemisphere, 34 species were recognized as valid in 1999 [4], and by 2015, 29 species had been documented from Europe, North Africa, and the Middle East [5]. While most *Stelis* species are assigned to *Stelis* s. str., six recognized subgenera exist to accommodate species with deviant morphological characters [1]. Among these, the subgenus *Stelidomorpha* was originally established as a distinct, monobasic genus [6] to accommodate *Stelis nasuta* (Latreille, 1809). This species is distinguished from other *Stelis* species known at that time by unique features, including an elongated, modified clypeus and a claw-like extension of the inner tooth on the fore tibia [7,8]. Apart from some taxa which are today no longer regarded as valid, *S. aegyptiaca* Radoszkowski, 1876, and *S. pentelica* Mavromoustakis, 1963, were added to this genus later [9,10]. *Stelidomorpha* is today recognized as a subgenus of *Stelis* [1,7,10,11,12,13,14]. In addition to the three species combined under the subgenus *Stelis* (*Stelidomorpha*), a fourth, still undescribed species of *Stelidomorpha* was mentioned in Kenya [1,15]. No further information is available on it.

Since a review of *Stelis* species from Europe, North Africa, and the Middle East published in 2015 [5], new data have significantly advanced our understanding of this subgenus. Through a genetic analysis of its members, we uncovered novel insights into infrageneric diversity and phylogeographic patterns. For one species, we identified distinct genetic lineages and used morphometric methods to further assess whether these lineages represent separate species. To complement this, we applied automatic species delimitation (ASD) methods—tools still rarely used in bee taxonomy—to further analyze the genetic data. As a result, we discovered two previously undescribed species from the Arabian Peninsula. In this study, we describe these new species, elevate one subspecies to species rank, present an updated identification key for the subgenus *Stelidomorpha*, and provide detailed distribution maps for all known species.

## 2. Materials and Methods

### 2.1. Photography

Photographs were taken with a Canon MP-E65/2.8 lens mounted on a Canon EOS 6D camera. The camera was moved between the shots with a Cognisys StackShot Rail and usually between 20 and 30 photographs were taken at different focal distances to give a resulting image with a greater depth of field than any of the individual source images. Subsequently, the pictures were processed with Helicon Focus (version 6.7.1) software to combine the pictures and to create one completely focused image from several partially focused images (image stacking). The resulting images were further processed with Adobe Photoshop Elements 15.

### 2.2. DNA Analysis

In order to support species identification and reveal phylogenetic relationships, the barcoding unit of the mitochondrial cytochrome c oxidase I (COI) gene of 27 specimens was examined. These include eleven sequences generated by M.K., nine sequences previously uploaded to BOLD by C. S.-E., and seven sequences publicly available in BOLD (https://www.boldsystems.org; downloaded on 5 August 2025) (Table 1).

DNA extraction, PCR amplification, and sequencing were conducted by the Canadian Centre for DNA Barcoding (CCDB), Guelph, using standardized high-throughput protocols (http://ccdb.ca/resources, accessed on 15 January 2025) [16]. DNA alignments were made with the Muscle Alignment, run on MEGA11 software [17]. Maximum Likelihood (ML) and Maximum Parsimony (MP) Tree phylogenetic analyses were also performed using the MEGA software [17,18], and bootstrap values were determined from 1000 replicates using the model which received the lowest BIC (Bayesian Information Criterion) scores in a comparative analysis of various models and is therefore considered to best describe the substitution pattern (Hasegawa-Kishino-Yano HKY or Tamura-Nei TN93 models). *Stelis ruficornis* Morawitz, 1872, and *S. signata* (Latreille, 1809) were chosen as the outgroup.

For *Stelis nasuta*, haplotype network analysis was performed using the PopART v1.7 (Population Analysis with Reticulate Trees) software, developed by the University of Otago (https://popart.maths.otago.ac.nz, accessed on 7 July 2025). The analysis employed Median Joining Network (MJN) methodology [19], which is particularly effective for studying populations with high genetic variability. MJN introduces “median vectors”—hypothetical ancestral or intermediate sequences—to better represent ambiguous evolutionary events. This approach is considered more suitable than the commonly used Minimum Spanning Network (MSN), which prioritizes simplicity by constructing a tree-like network with minimal or no reticulations, potentially overlooking complex evolutionary relationships.

### 2.3. Molecular Species Delimitation

To delimit Operational Taxonomic Units (OTUs) for *Stelis nasuta*, we adopted the phylogenetic species concept based on COI sequences. Species boundaries were estimated, and the number of putative species within the material was assessed using four different single-locus species delimitation methods.

We used the distance-based Automatic Barcode Gap Discovery (ABGD) to identify putative species by detecting a “barcode gap” that separates intraspecific from interspecific genetic variation [20]. Additionally, we applied the Assemble Species by Automatic Partitioning (ASAP) program, an advancement of ABGD, that also clusters sequences into putative species using pairwise genetic distances. Unlike ABGD, ASAP does not require predefined thresholds, instead ranking partitions with a scoring system to identify the best-supported species boundaries [21]. For both models, pairwise genetic distances were computed using the Jukes–Cantor (JC69) model. Analyses were performed on the online SPART platform https://spartexplorer.mnhn.fr (accessed on 10 August 2025) [22]. In ABGD, the relative gap width was chosen as 1.5, and the minimum and maximum a priori values as 0.001 and 0.1. ASAP was set to split groups at a probability below 0.001. The Bayesian Poisson Tree Process (bPTP) approach, which infers putative species boundaries from a phylogenetic input tree by analyzing branch lengths [23], assumes that the number of substitutions between species significantly exceeds the number within species. For our analysis, we used the bPTP program (downloaded from https://itaxotools.org on 10 August 2025) with a ML tree rooted on midpoint, obtained from MEGA11 and stored in Newick format as input. The analysis was conducted using default *p*-values. As an additional method, we employed the Refined Single Linkage (RESL) system, which integrates single linkage clustering—grouping sequences based on pairwise genetic distances—with subsequent Markov Clustering [24]. RESL is a built-in function of the BOLD system (https://www.boldsystems.org, accessed on 15 January 2025) and provides output in the form of Barcode Index Numbers (BINs).

### 2.4. Distribution

The distribution pattern of *Stelis nasuta* was analyzed using four different data sources: (1) Literature data. A comprehensive literature survey revealed approximately 150 records, of which 130 were precise enough to determine geographic coordinates. This was performed with Google Earth Pro, version 7.3.6.9796. (2) Collection data. A total of 192 occurrence records were obtained from collections, with the majority from the Anthidiini collection of M.K. (CMK) (178 records, 92%). Additional material included material from L. Bertsch (1), N. Benarfa (2), P. Geisendörfer (1), W.-H. Liebig (1), and T. J. Wood (6). Records were also sourced from institutions such as the Biodiversity Centre Linz, Austria (1), the Deutsches Entomologisches Institut, Eberswalde, Germany (1), the Natural History Museum, University of Tartu, Estonia (1), and the Laboratory of Zoology, University of Mons, Belgium (1) (see acknowledgements for details). (3) GBIF data: A total of 286 occurrence records were retrieved from the Global Biodiversity Information Facility, GBIF [25], drawing data from the databases of 10 organizations. The Swiss National Apoidea Databank was the main contributor, providing 160 records. After supplementing missing geographic coordinates and filtering out incomplete records, 273 reliable occurrence records were included in the analysis. (4) PULSE Project Database: This EU-funded project, implemented by the University of Mons, Belgium, provides technical and scientific support for monitoring European biodiversity. The PULSE database contains 449 specimen records, most of which were retrieved from other sources, including GBIF. All data from sources 1–4 are compiled in the Supplementary Annex. While there are significant overlaps among these sources, the absence of unique identifiers for occurrence data made the removal of duplicates unfeasible. However, duplicate geographic coordinates are overlaid on the distribution map, ensuring that they do not affect its quality. Distribution maps were generated using SimpleMappr v3.1 [26].

### 2.5. Morphometric Analysis

For a morphometric analysis, the following eleven lengths of the head and one length of the scutum and one of the wings were measured following the methodology described in [27,28,29]: eye length; eye width; head width; eye distance; interantennal distance; vertex length; ocellus–eye distance; hind ocelli distance; hind ocellus–fore ocellus distance; marginal cell length; and scutellum length. The relevant body parts were photographed for this purpose and the measurements were taken from the photograph. A multivariate Linear Discriminant Function Analysis (LDA) was performed with PAST, version 4.15 [30], to test differences between groups. The methodological approach was described in more detail elsewhere [27,28,29].

## 3. Results

***Stelis*, Subgenus *Stelidomorpha***

Members of the genus *Stelis* are distinguished from the other genera of Anthidiini by the presence of arolia, two apical spines on both the fore- and mid-tibiae, tegulae of ordinary size, and the absence of a scopa in females. The subgenus *Stelis* (*Stelidomorpha*) is characterized by a prolonged clypeus that overhangs the mandibles, and a long, claw-like inner tooth at the apex of the fore and mid tibiae. A full characterization of the genus and its subgenera is provided in [1,4].

**Figure 1:**
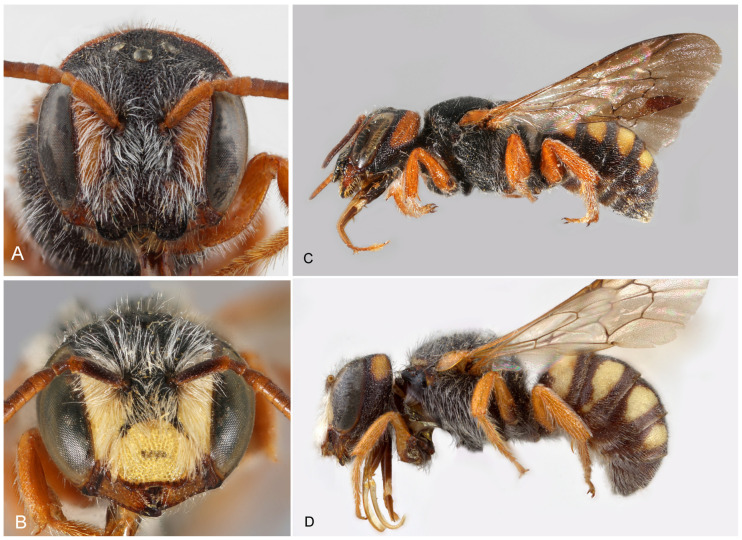
*Stelis aegyptiaca* (Radoszkowski, 1876). Nominate subspecies. (**A**,**C**) Female; (**B**,**D**) male; Female (**A**) from Egypt, (**C**) from Morocco; Male (**B**) from Morocco, (**D**) from Egypt.

***Stelis aegyptiaca* (Radoszkowski, 1876)**
  (Figure 1)

*Stelidomorpha aegyptiaca* Radoszkowski, 1876.—Egypt [9].

*Stelis vachali* Pérez, 1895.—Tunisia. Synonymy by Warncke (1992) [13]. Holotype in Muséum national d’Histoire naturelle, Paris.

*Stelis thebaidis* Friese, 1899.—Egypt [14]. Synonymy by Noskiewicz (1961) [8]. Holotype in Oxford University Museum of Natural History.

*Barcode Index Number (BIN)*: BOLD:AFC0429 (based on two specimens from Egypt).

*Material examined*. ALGERIA: 13 ex.; Ain Zaatout (35.14° N 5.83° E); 26.v.1971 [31], 1 ex. examined in DEI.—EGYPT: 8♂; Edfou (24.97° N 32.87° E); 20.–22.11.1958; W. J. Puławski leg. (CMK).—1♀ 5♂; Luxor (25.68° N 32.63° E); 24.–26.ii.1958; W. J. Puławski leg. (CMK).—1♀; Wadi Digla (29.94° N 31.37° E); 16.iii.1938; H. Priesner leg. (CMK).—ISRAEL: 1♂; Eilat (29.55° N 34.95° E); 06.iv.1973; M. Kaplan leg. (coll. Kaplan).—2♀; Wadi Segur, 40 km NW Eilat (29.91° N 34.87° E); 17.iv.1990; K. Warncke leg. (CMK).—JORDAN: Wadi Rum (29.55° N 35.40° E); 10.iv.1989, J. Gusenleitner leg.; 04–05.v.1996, Mi. Halada leg. (CMK).—LIBYA: Tripolitania: Jebel Soda [Jabal As-Sawda], Wadi Ghodaifa (28.67° N 15.82° E); 03.ii.1952 (CMK).—MOROCCO: Akka, 50 km SW (29.10° N–8.66° E); 430 m; 27.iii.1986; M. Schwarz leg. (CMK).—Drâa-Tafilalet, Midelt, R503, 7 km NR Ait Ben Yacoub (33.29° N–4.68° E); 21.v.2022; T. Wood leg. (CMK).—Drâa-Tafilalet, Tazenakht, 9 km W Anezal (30.77° N–7.36° E); c. 1600 m; 16.iv.2022; T. Wood leg. (CMK).—1♀ 3♂; Foum Zguid, 30 km N (30.34° N–6.84° E); 30.iii.1986; M. Schwarz leg. (CMK).—1♂; Errachidia, 5 km N Ammer, Oued Reg (31.92° N–4.43° E); 08.iii.1997; R. Wahis leg. (CSE).—TUNISIA: 1♀ 1♂; 10 km SE Foum Tataouine (32.85° N 10.50° E); 25.iii.2001; Ch. Schmid-Egger leg. (CSE).

Other material (literature data). ALGERIA: Biskra (34.85° N 5.72° E), as *S. vachali*; 29.iii.1897 and 13.v.1897 [31,32].—EGYPT: Edfou [=Edfu], 1958, W. J. Puławski leg. [32,33]. See also under material examined.—Luxor, type of *S. thebaidis* in Oxford Mus.; 18.iii.1899 [8].—MOROCCO: Drâa-Tafilalet, Jebel Bani (29.933° N–5.632° E) [34].—TUNISIA: Foum Tataouine (32.92° N 10.45° E) [35].

*Diagnosis/description*. Detailed, illustrated descriptions are available in the literature [4,7,28]. *Stelis aegyptiaca* is distinguished from *S. canaria* from the Canary Islands by lateral bands on T1 (uninterrupted band in *S. canaria*) and its yellow (not red) maculations (Figure 1).

**Figure 2:**
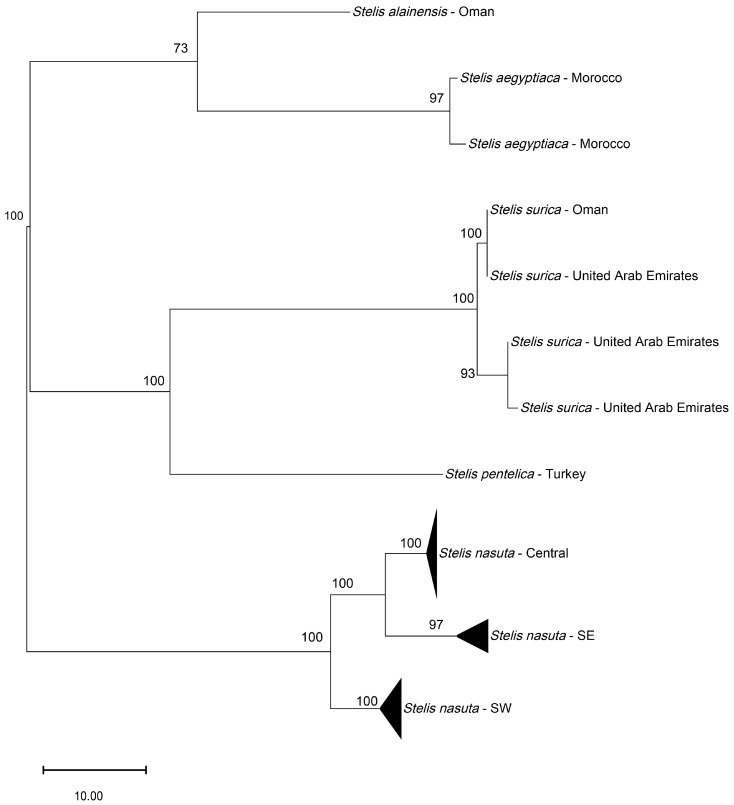
Species ID Tree (“phylogenetic tree”) of *Stelis*, subgenus *Stelidomorpha*, based on the barcoding unit of the mitochondrial COI gene. The evolutionary history was inferred using the Maximum Parsimony method. For *Stelis nasuta*, the DNA sequences of the three Operational Taxonomic Units (OTUs) have been collapsed.

**Figure 3:**
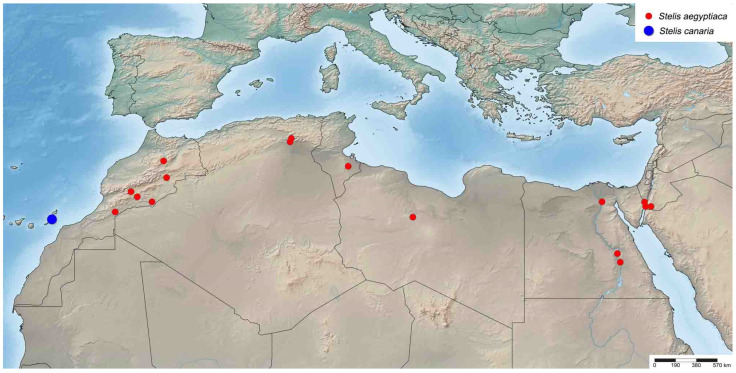
Distribution of *Stelis aegyptiaca* (Radoszkowski, 1876) and *S. canaria* Warncke, 1992.

*Genetic analysis*. Based on the COI gene sequences, *S. alainensis* sp. nov. is the closest relative among the species for which barcodes are available (Figure 2), with genetic distances ranging from 7.6 to 8.1%, followed by *S. pentelica*, which shows a distance of 9.5%. Genetic data of *S. canaria*, apparently the closest relative of *S. aegyptiaca* (formerly treated as its subspecies), are not available.

*Distribution*. The species is widely distributed in northern Africa from Morocco to the southern Levant (Figure 3). Not reliably reported from Turkey cf. [5]. A specimen from the UAE, originally published as *S. aegyptiaca* [36], was revealed herein to belong to *S. surica* sp. nov. Countries: Algeria, Egypt, Israel, Jordan, Libya, Morocco, and Tunisia.

**Figure 4:**
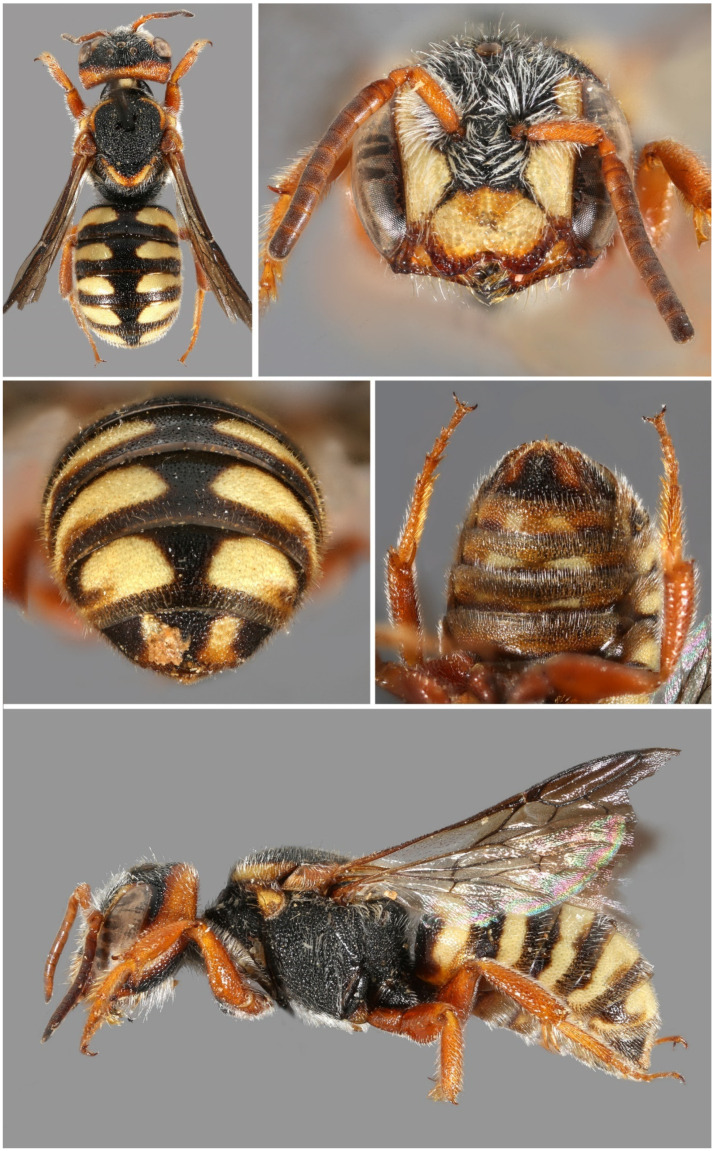
*Stelis alainensis* Kasparek sp. nov. Holotype, female from Oman.

**Figure 5:**
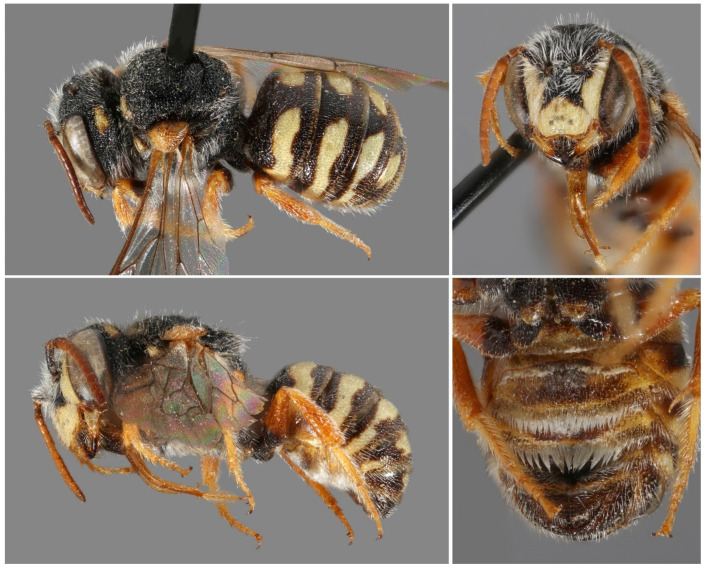
*Stelis alainensis* Kasparek sp. nov. Paratype, male from the United Arab Emirates.

**Figure 6:**
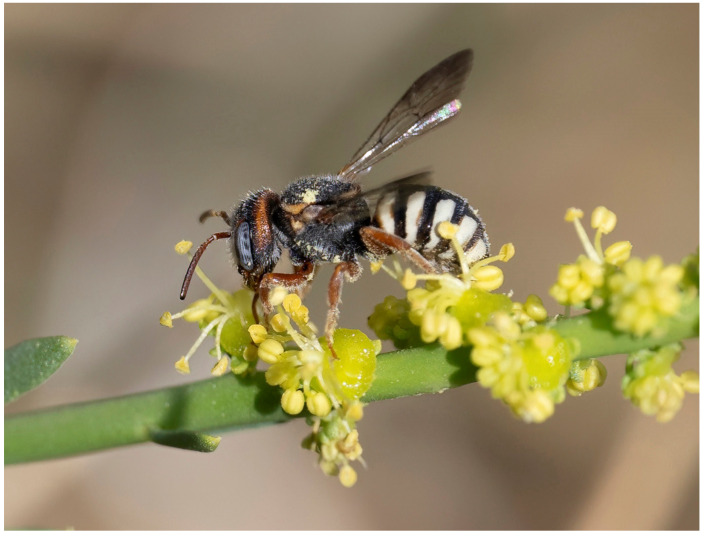
*Stelis alainensis* Kasparek sp. nov. on *Ochradenus arabicus* at Jebel Hafeet, United Arab Emirates, in March 2023. Photograph: H. Roberts.

***Stelis alainensis* Kasparek sp. nov.**
  (Figure 4, Figure 5 and Figure 6)

*Barcode Index Number (BIN)*: Not yet available. 

*Material*. HOLOTYPE: Female; Oman: 7 km S of Mahda (24.348° N 55.978° E), 17.iii.2022, leg. M. Halada leg. (CMK, mk0776).—PARATYPES: 1♀; United Arab Emirates (UAE): Ain Al Waal, Al Ain, Jebel Hafeet (24.07° N 55.75° E), 05.iii.2023; leg. H. Roberts (coll. H. Roberts, hr015).—2♂; UAE: Jebel Hafit [=Jebel Hafeet] (24.07° N 55.75° E); 11.–19.iii.2009; leg. Ch. Schmid-Egger (CSE).

*Diagnosis*. The female is characterized by a predominantly yellow clypeus, a trait shared within the subgenus only with *S. pentelica* and *S. surica* sp. nov. It can be distinguished from *S. pentelica* by features such as the apically rounded scutellum (shallowly emarginate in *S. pentelica*), and the shiny, shallowly punctate terga (e.g., punctures on T1 mostly with a distance of one puncture diameter, whereas the punctures in *S. pentelica* are deeper with narrow ridges in between). The male of *S. alainensis* sp. nov. is characterized by orange-brown antenna (except for the black scape). It is distinguished from *S. pentelica* by the widely rounded posterior margin of the scutellum (V-shaped in *S. pentelica*), from *S. aegyptiaca canaria* by the pale-yellow maculation of the terga (orange-red in *S. aegyptiaca canaria*), and from *S. a. aegyptiaca* by the shape and size of the tergal maculae (light tergal bands on T1–T3 reaching the lateral margin in *S. alainensis* sp. nov., not reaching in *S. a. aegyptiaca*).

*Description. Female*. Body length: 8.0–8.5 mm. Intertegular length 1.95 mm (holotype) and 2.06 mm (paratype); marginal cell length: 1.63 mm (holotype) and 1.91 mm (paratype).—*Head*: Black with pale yellow maculation in the face and an orange-red preoccipital band. Clypeus broad, bilobed, widely overhanging mandibles; pale yellow base with a narrow black margin, apical margin with broad dark brown band; pale yellow areas with some faded orange stains; punctation irregular and coarse with confluent punctures; paraocular area pale yellow, narrower in the upper than in the lower paraocular area; preoccipital band entire, reaching mandibular article, and covering almost entire gena; orange maculation linked with upper paraocular area in the paratype. Mandible dark orange-red at base and shiny red-brown teeth; one strong apical tooth and a less strong inner tooth (two teeth fused together?). Antenna red-brown, with the scape and the inner 3–4 flagellomeres brighter than the distal ones.—*Mesosoma*: Scutum black with yellow anterolateral band (margins of the band reddish-brown); width of scutum tapering towards posterior; scutellum and axillae semi-triangular in dorsal view; yellow band with some red-brown stain on outer side; margin with transparent lamella and a crown of white hairs; pronotal lobe large, yellow (darker on inner side), angular laterally and with a high lamella anteriorly; legs dark orange.—*Metasoma*: Terga black with large pale yellow lateral spots not reaching the midline; apical margin of T6 erose; shiny; punctation of terga fine, with distance between punctures mostly 1–2 puncture diameters; marginal zone transparent brown. Sterna with yellow and brown transversal maculations; S6 semicircular, somewhat elevated in the middle.

*Male*. Body length: 5.5–6.5 mm. Intertegular length 1.55–1.81 mm, marginal cell length 1.21–1.58 mm.—*Head*: Black with yellow paraocular area, clypeus and preoccipital band. Minute yellow spot in the supraclypeal area. Apical clypeal rim translucent light brown. Mandible translucent light brown with three black teeth. Preoccipital band reaching mid of eye; broad at ventral end, tapering upwards; interrupted in the middle. Preoccipital band in one male reduced to yellow spots behind the eye. Antenna reddish-brown, scape dark brown to black.—*Mesosoma*: Scutum black, convex; large but shallow punctures, separated by narrow ridges; yellow band along anterior margin. Pronotal lobe yellow (with dark base in one specimen) with anterolateral lamella. Outer margin of scutellum widely rounded; large punctures, almost double the size of the punctures on scutum. Punctation of axilla finer. Yellow band on outer side of scutellum, interrupted in the middle (only small remnants of the band in one specimen). Legs orange-brown with black bases of femora. *Metasoma*: T1–T5 (T6) with broad lateral light-yellow bands reaching the lateral margin at their contacts with their corresponding sterna. Light maculae laterally with brown inclusions. T7 retracted and hidden under T6, only black minute median tooth visible. S2 and S3 with white fringe of hairs.

*Genetic analysis*. Through DNA barcoding, a DNA fragment of the COI gene of the female holotype was obtained with 483 nucleotides. DNA could not successfully be retrieved from other specimens. The DNA sequence confirms the identity of the material as belonging to a distinct species, closely related to *Stelis aegyptiaca* (genetic distances 7.6–8.1%), *S. pentelica* (9.5%), *S. nasuta* (11.4–13.2%), and *S. surica* sp. nov. (11.9–12.7%) (Figure 2).

*Derivatio nominis*. The specific epithet is derived from “Al Ain”, a city in the Emirate of Abu Dhabi, United Arab Emirates, and close to the border with Oman. The Al Ain area is the type locality of *Stelis alainensis* sp. nov. Al Ain literally means “The Spring” and the town is known as the “Garden City” in the Gulf region.

**Figure 7:**
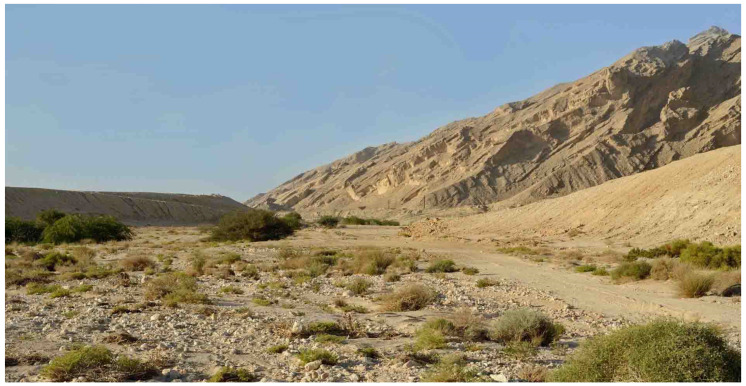
Jebel Hafeet in the United Arab Emirates, habitat of *Stelis alainensis* Kasparek sp. nov.

*Biology*. Found on the wing in early spring between early March and early April. At Jebel Hafeet (UAE) (Figure 6 and Figure 7), adults were recorded flying at *Ochradenus arabicus* Chaudhary, Hillc. & A.G. Mill, *Tephrosia purpurea apollinea* (Delile) Hosni & El-Karemy, and *Taverniera cuneifolia* (Roth) Arn.

Potential hosts for this cleptoparasitic bee species at Jebel Hafeet include 15 members of the family Megachilidae that have been recorded within 200 m of *S. alainensis* sp. nov. collection sites and within the same season, albeit in different years. These were as follows: *Anthidium minimum* Pasteels, 1969, *A. tesselatum* (Klug, 1832), *Eoanthidium crenulatum* (Warncke, 1982), *Hoplitis hofferi* (Tkalců, 1977), *Icteranthidium* aff. *ferrugineum* (Fabricius, 1787), *Megachile (Chalicodoma)* cf. *atrocastanea* (Alfken, 1932), *M. (Chalicodoma) maxillosa* (Guérin-Méneville, 1845), *M. (Eutricharaea) leucostoma* (Pérez, 1907), *M. (Eurymella) patellimana* (Spinola, 1838), *M. (Eutricharaea)* cf. *leachella* (Curtis, 1828), *M. (Eutricharaea) rufomandibularis* (Praz, 2021), *M. (Eutricharaea) soikai* (Benoist, 1961), *M. (Eutricharaea) walkeri* (Dalla Torre, 1896), *M. (Pseudomegachile) flavipes* (Spinola, 1838), and *Pseudoanthidium guichardi* (Pasteels, 1980).

**Figure 8:**
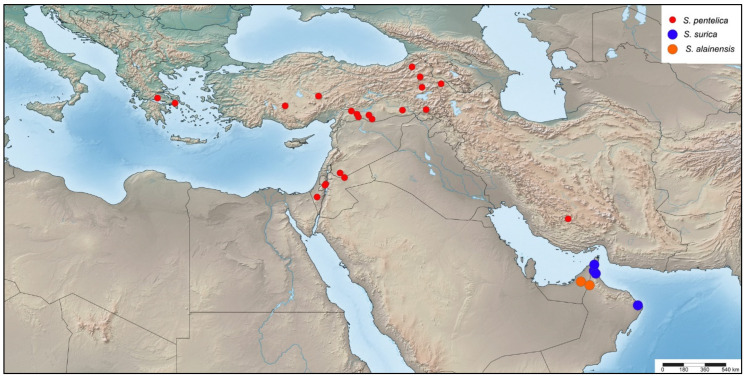
Distribution of *Stelis alainensis* Kasparek sp. nov. (orange dots), *S. pentelica* Mavromoustakis, 1963 (red dots), and *S. surica* Kasparek sp. nov. (blue dots).

*Distribution*. Known from Oman and the United Arab Emirates (UAE) (Figure 8). Among the UAE records of this species were adult individuals photographed at Jebel Hafeet (Figure 6), a mountain that spans the border between the UAE and Oman. This isolated anticlinal massif rises sharply to over 1140 metres above the surrounding flat terrain south of Al Ain, Abu Dhabi. Specimens were observed on mountainside plants at elevations of approximately 250–300 m.

**Figure 9:**
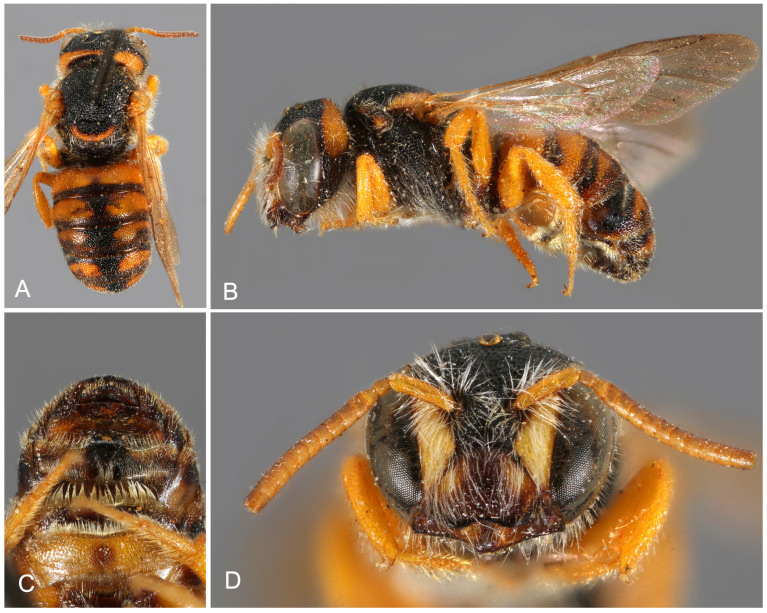
*Stelis canaria* Warncke, 1992, **stat. nov.** Holotype, male. (**A**) Habitus dorsal. (**B**) Habitus lateral. (**C**). Metasoma ventral. (**D**). Head frontal.

***Stelis canaria* Warncke, 1992 stat. nov.**
  (Figure 9)

*Stelis aegyptiaca* ssp. *canaria* Warncke, 1992.—Spain: Canary Islands. Holotype in CMK.

*Stelis aegyptiaca* ssp. *fuerteventurae* Tkalců, 1993.—Spain: Canary Islands [37]. Synonymy by Tkalců (1993), footnote on p. 858 [37]. Holotype in Museo Insular de Ciencias Naturales de Tenerife, Santa Cruz de Tenerife.

*Material examined*. HOLOTYPUS. SPAIN (Canary Islands): Fuerteventura (28.49° N–13.86° E); 13–23.iii.1926; ex coll. Warncke (CMK).—Betancuria, Fuerteventura (28.42° N–14.05° E); 11.–13.iii.1984; H. Teunissen leg. (CMK).—1♀; Puerto del Rosario, Fuerteventura (28.49° N–13.86° E); 04.–16.iii.1948 [1984?]; H. Teunissen leg. (CMK).

*Other material*. SPAIN: Fuerteventura, 14 km W Puerto del Rosario; 7.iv.1982; leg. H. Hohmann (Museo Insular de Ciencias Naturales de Tenerife, Sta. Cruz de Tenerife) [34].—Fuerteventura, Vallebrón, 12 km NW Puerto del Rosario, 24.iv.1984; leg. H. Hohmann (coll. Übersee-Museum Bremen) [37].

*Diagnosis/Description*. Detailed descriptions are available in the literature [13,37]. *Stelis canaria* can be distinguished from *S. aegyptiaca* by the uninterrupted band on T1 (often also on T2), whereas *S. aegyptiaca* exhibits lateral bands that do not reach the midline. Additionally, all maculations are red-brown in *S. canaria*, compared to yellow in *S. aegyptiaca*. Examination of larger series of specimens previously assigned to *S. aegyptiaca* has confirmed these traits as consistent diagnostic characters, thereby justifying the elevation of *S. aegyptiaca canaria* to species rank.

*Distribution*. Endemic to the Canary Islands, where it has so far only been found on Fuerteventura (Figure 3).

**Figure 10:**
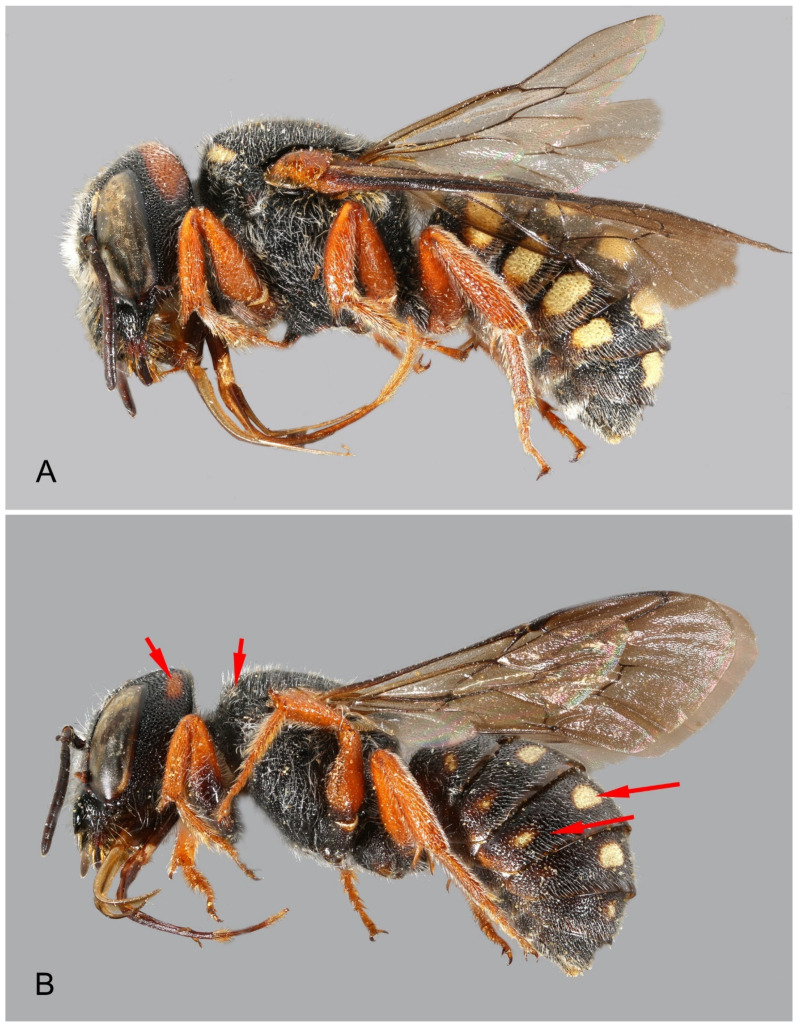
*Stelis nasuta* (Latreille, 1809), female, from Turkey (**A**) and Morocco (**B**). The two females belong to different genetic Operational Taxonomic Units (OTUs). However, differences in colouration such as the extent of the yellow pattern shown on the photographs (see arrows) are not fully diagnostic.

***Stelis nasuta* (Latreille, 1809)**
  (Figure 10)

*Anthidium nasutum* Latreille, 1809.—France, Paris (Meudon).

*Anthidium nasale* Latreille, 1809, incorrect secondary spelling, *Stelidomorpha nasuta* (Latreille, 1809) [38].

**Figure 11:**
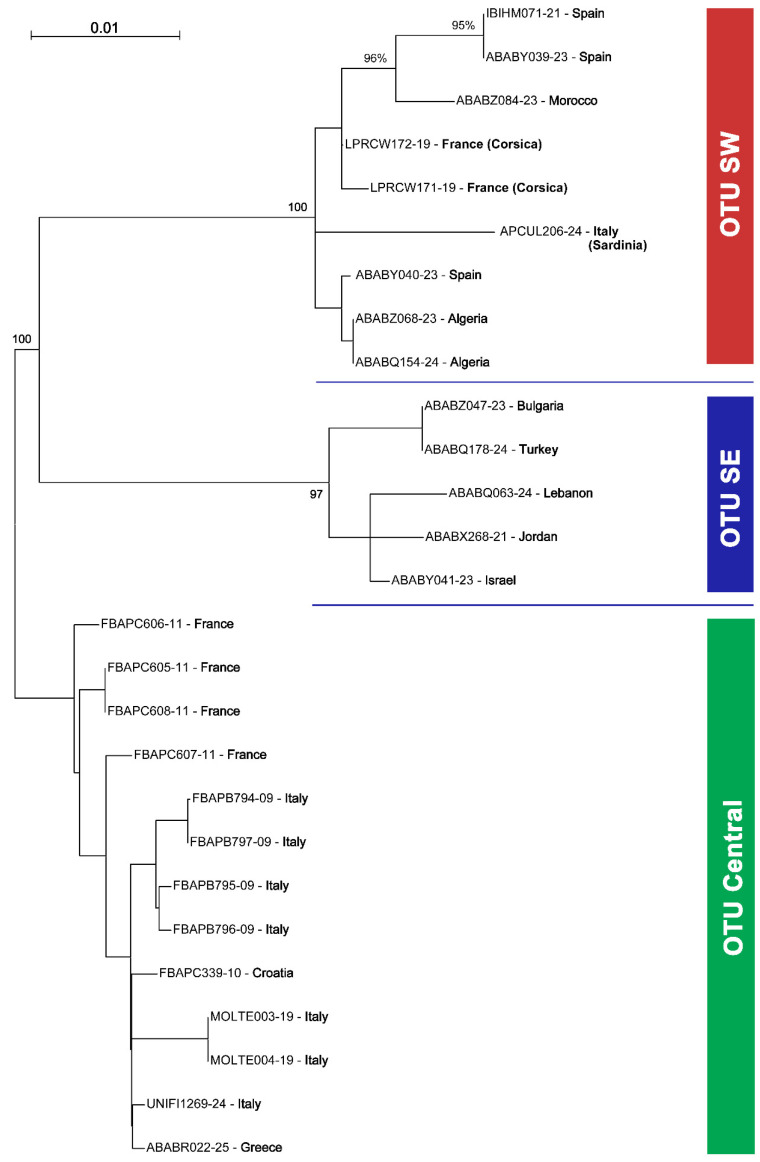
Phylogenetic Maximum Likelihood (ML) Tree for *Stelis nasuta* s.l. showing the three main lineages (Operational Taxonomic Units, OTUs) in the West Palaearctic, based on the Tamura 3-parameter model. The tree is rooted on OTU Central and bootstrap values are shown for the main nodes.

**Figure 12:**
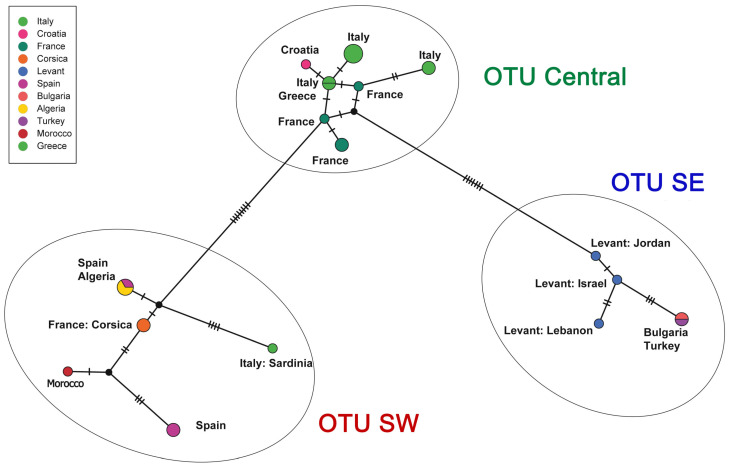
Haplotype network analysis of *Stelis nasuta* s.l. Median-joining networks based on the barcoding fragment of the cytochrome oxidase I (COI). Each sequenced haplotype is represented by a circle, the size of which is proportional to its overall frequency in the dataset. Black lines and hatch marks on the branches are used to indicate the number of mutational changes between two different haplotypes. The black dots represent median vectors—hypothetical ancestral haplotypes that are inferred to explain the evolutionary paths among observed haplotypes. The colours correspond to the countries in which the material was sampled.

**Figure 13:**
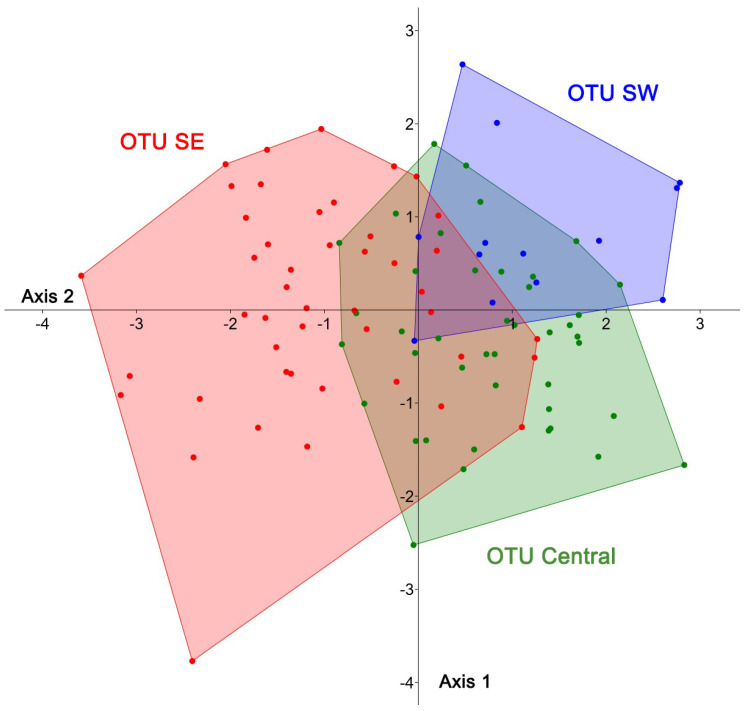
Results of a Linear Discriminant Function Analysis (LDA) carried out for putative members of the three Operational Taxonomic Units (OTUs) of females of *Stelis nasuta* s.l. The OTUs were identified through genetic barcoding (see Figure 11 and Figure 12).

*Barcode Index Numbers (BINs)*: BOLD:AAO3679 for OTU Central, BOLD:AEC4732 for OTU SW, and BOLD:ABW1388 for OTU SE (Figure 10, Figure 11, Figure 12 and Figure 13).

*Diagnosis/Description*: Description with drawings and photographs available in the literature [5].

*Genetic analysis*. A total of 27 DNA sequences from 12 countries were available for this study: Algeria (2), Bulgaria (1), Croatia (1), France (4 mainland, 2 Corsica), Greece (1), Israel (1), Italy (6 mainland, 1 Sardinia), Jordan (1), Lebanon (1), Morocco (1), Spain (3), and Turkey (1) (Table 1). For the further analysis, only sequences >560 bp were used, i.e., two sequences from Italy were disregarded in the species delimitation analysis.

A haplotype analysis found a nucleotide diversity of π = 0.0189, with 41 segregating and 31 parsimony-informative sites, and resulting in a slightly negative Tajima’s D (D = −0.133), which is not significant (*p* > 0.05). The relatively high nucleotide diversity may suggest a genetically diverse population, a large effective population size, or an admixture of various lineages. The slightly negative Tajima’s D generally suggests a mild excess of rare alleles, but as the value is not statistically significant, no further interpretations are possible.

A ML phylogenetic tree (Figure 11) of 27 COI sequences revealed that what has been assigned to *S. nasuta* consists of three distinct Operational Taxonomic Units (OTUs) (Figure 10, Figure 11, Figure 12 and Figure 13). The first OTU consists of sequences from southwestern France, Italy, Croatia, and Greece (OTU Central). A second OTU includes sequences from Iberia and northern Africa (Morocco, Algeria) but also sequences from the islands of Corsica and Sardinia (OTU SW). A third OTU is composed of sequences from the southern Balkan (Bulgaria), Turkey, and the Levant (Israel, Jordan, Lebanon) (OTU SE). A haplotype network analysis (Figure 12) confirmed the existence of these three clusters. There are no haplotypes shared among these three clusters. The haplotype network also shows that there is no direct link between OTU SW and OTU SE, but they are connected via OTU Central.

*Morphological and morphometric analysis*. A morphological comparison including an analysis of the colour of the integument and the terminalia of these three groups did not reveal differences. For a morphometric analysis, 13 length traits of the wing, the mesosoma, and the head were examined. To enable a comparison, they were clustered in the three OTUs identified by genetic barcoding (Figure 12 and Figure 13). A Principal Component Analysis (PCA) identified 11 principal components, with the first component alone explaining 94.2% of the total variance, and the first two components together accounting for 95.9%. PC1 was driven mainly by head width, followed by length of the marginal cell and eye length, whereas PC2 was negatively influenced by marginal cell length. The scatterplot revealed no obvious groupings, and plots of PC1 against the other components showed largely overlapping convex hulls for the three OTUs, except for the fact that OTU SE displayed a higher variance. Also, the reduction in parameters, achieved by removing less relevant ones, did not produce any clearly discernible groups. In a Linear Discriminant Function Analysis (LDA), these three groups were incompletely separated, and all show large overlap of the combined morphological traits (Figure 13). The convex hull (polygon) of the OTU-SE was revealed to be larger than the other two hulls. This higher morphometric variation may be a consequence of geographical variation within the distribution area, which is, in this OTU, larger than in the other two species.

**Figure 14:**
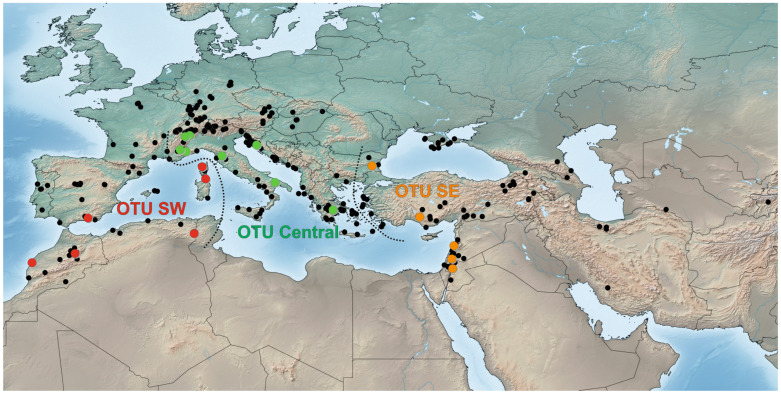
Distribution of *Stelis nasuta* (Latreille, 1809). Black dots represent occurrence records based on published and unpublished sources (available in the Supplementary Annex). Coloured dots show the members of three distinct phylogenetic lines identified by genetic barcoding (OTUs: blue = SW lineage, green = central lineage, and red = SE lineage). The dotted lines indicate the approximate borders between these Operational Taxonomic Units, and the delimitation used for morphometric analysis.

*Distribution*. *Stelis nasuta* exhibits a wide distribution, spanning from the Atlantic region (including Morocco and the Iberian Peninsula) over the Mediterranean to Central Asia (Figure 14). Its occurrence in Uzbekistan and Tajikistan, previously noted without concrete records [13,39], has been confirmed through material housed in the CMK collection. However, no direct evidence is available for its presence in Iraq and Kazakhstan beyond a generic reference [39], and these records require further confirmation. A historical record from Sweden [40] is considered unreliable, as already noted in the literature [41].

Over the past century, the northern range limit of *S. nasuta* is known to have shifted southward. The species has disappeared from several northern regions, including northern and western France (the species was originally described from Meudon, Paris, more than 200 years ago!), southern Germany, and parts of northern Switzerland [38,42].

OTU SW is primarily found in northern Africa (Algeria and Morocco) and the Iberian Peninsula. In France, populations in the subalpine regions of the southeast belong to OTU Central, while it is likely that populations in other parts of France belong to OTU SW, an assumption based on distribution patterns observed in other anthidiine bees (M.K., unpublished). Also, the (extinct) populations in Germany may belong to OTU SW. Surprisingly, specimens from Corsica and Sardinia have also been attributed to OTU SW. The confirmed range of OTU Central stretches from the Alpes-Maritimes in France through Italy into the Balkans. The status of the population in Sicily remains uncertain but is likely to belong to OTU Central. The distribution of OTU Central in the Balkans apparently includes eastern Europe and extends in the south as far as Greece. It is postulated here that the mid-Aegean trench, which is an important barrier in many species including Anthidiini [43,44], separates OTU Central from OTU SE. OTU SE encompasses populations in Bulgaria, Turkey, and the Levant, with records from Central Asia likely belonging to this OTU as well. Overall, the three identified megapopulations of *S. nasuta* exhibit clearly defined core distribution areas. Nevertheless, the precise boundaries between these populations and possible local overlaps require further investigation to fully elucidate their ranges.

**Figure 15:**
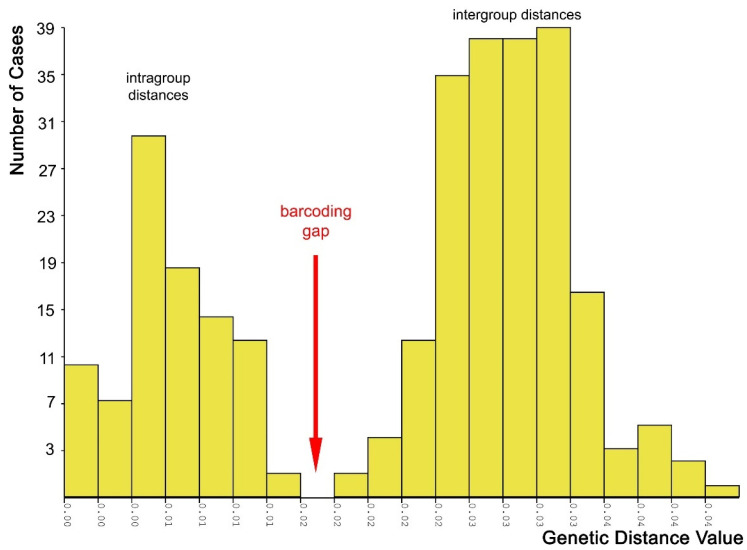
Pairwise genetic distances among the barcoding unit of the COI gene in the megapopulation of *Stelis nasuta* s.l., showing the intragroup and intergroup variations. The graph is based on 25 sequences with >560 bp from throughout the species’ distribution area. The graph was generated with ABGD, while its design was modified.

*Molecular species delimitation*: Both the species ID tree (Figure 2 and Figure 11) and the haplotype network (Figure 12) based on the barcoding unit of the COI gene show three distinct clusters of DNA sequences, and the members of these three clusters are found in geographically clearly delimited areas (see above). At the same time, the analysis of the pairwise genetic distances revealed a barcoding gap clearly delineating inter- and intraspecific variation (Figure 15), i.e., strong evidence that the sample consists of more than one species. This is supported by a high nucleotide diversity which may be explained by a sample containing members of more than one lineage.

**Figure 16:**
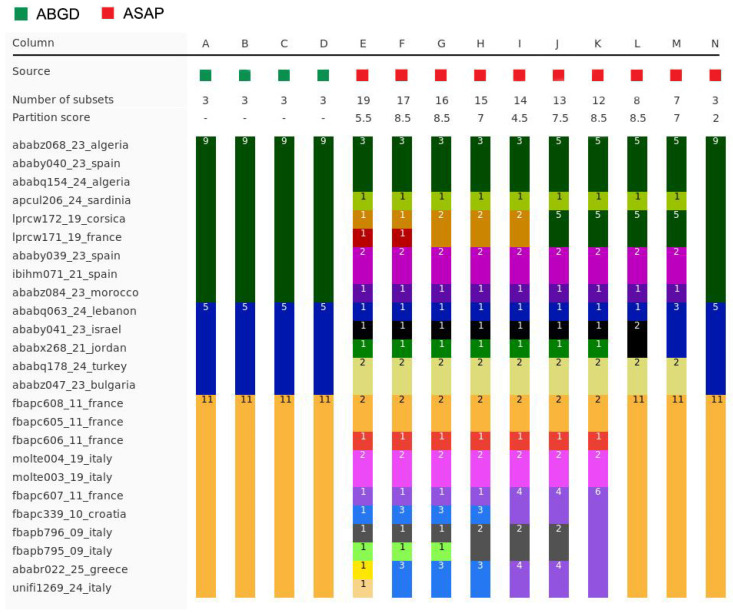
Results of partitioning the sequences of the genetic barcoding unit of the COI DNA sequence of *Stelis nasuta* s.l. into putative species. The graph is based on 25 DNA sequences with >560 bp from throughout the species’ distribution area. The graph was produced with the Spart Explorer web platform and shows species partitions produced by ABGD and ASAP. ASAP shows the partitions with the 10 best ASAP scores, wherein the lower the score, the higher the rank. The different partitions are represented by distinct colours on the bar, and the number inside corresponds to the number of assigned specimens.

The ranked-distances graph of the ABGD analysis reveals a stable plateau between *p* = 0.0010 and 0.0046, during which three groups are consistently recovered. This pattern indicates the presence of a strong barcode gap, supporting the delimitation of three putative species or Operational Taxonomic Units (OTUs). These groups correspond exactly to the clusters identified in the haplotype network, namely OTU Central, OTU SW, and OTU SE (Figure 11, Figure 12 and Figure 16). Using ASAP, we calculated the 20 best-scoring partitions, on the basis of split groups at divergence levels below 0.001. The lowest ASAP score was 2.0, which represents the best-supported partition, and it also recovered the same three clusters of putative species as ABGD. In parallel, the bPTP analysis based on the ML phylogeny likewise identified three putative species across the full dataset. These species assignments matched the results from ABGD and ASAP, as well as the clusters identified in the haplotype network. Additionally, bPTP suggested the possibility of a more refined clustering of the DNA sequences, indicating possible sub-clustering within one or more of the groups. Finally, the RESL algorithm, integrated in the BOLD System, also divided the sequences into three groups that align perfectly with the three OTUs. Specifically, RESL assigned the following Barcode Index Numbers (BINs) to these groups: BOLD:AAO3679 to OTU Central, BOLD:AEC4732 to OTU SW, and BOLD:ABW1388 to OTU SE.

***Stelis pentelica* Mavromoustakis, 1963**
*Stelis pentelica* Mavromoustakis, 1963.—Greece.
*Stelis bicornuta* Pasteels, 1969.—Synonymy by Warncke (1992) [13].

*Barcode Index Number (BIN)*. BOLD:AGP9783 (based on specimen RMNH.INS.1662642 from Turkey derived from the BOLD database).

*Material examined*. GREECE: 2♀; Parnaß, 5 km W Arachova (38.48° N 22.52° E), 1300 m; 24.vii.1984; M. Hassler leg. (CMK).—IRAN: 1♀; Fars, 20 km W Neyriz (29.18° N 54.10° E), 1550 m; 18.v.1978; K. Warncke leg. (CMK).—PALESTINE: 1♂; Jericho (31.86° N 35.46° E); 12.iv.1941; Bytinski-Salz leg. (CMK).—2♀; Wadi Qilt Ein-el-Fawwar (31.77° N 35.40° E); 01.vi.1991; K. Warncke leg. (CMK).—SYRIA: 1♀ 1♂; Kafr, Suwayda (32.71° N 36.56° E); 21.vi.2000; M. Halada leg. (CMK).—1♂; Anata, 50 km SE of Suwayda (32.36° N 36.90° E); 20.–21.v.1996; Mi. Halada leg. (CMK).—TURKEY: 1♀; Ağrı: 20 km N Patnos (39.31° N 42.86° E), 1650 m; 09.viii.1982; K. Warncke leg. (CMK); 1♂; Ağrı: Ararat southern slope (39.58° N 44.33° E), 1800 m; 02.–03.vii.1985; M. Schwarz leg. (CMK).—1♀; Hakkâri: Suvari-Halil Pass E of Beytüşşebap (37.59° N 43.18° E), 2300 m; 02.viii.1982; K. Warncke leg. (CMK).—1♂; Kahramanmaraş: Pazarcık-Ganidağı (37.48° N 37.43° E), 1000 m; 11.vi.2008; M. Kafka leg. (CMK).—2♀; Kars: Karakurt (40.11° N 42.72° E), 08.viii.1979; K. Warncke leg. (CMK).—1♀; Konya: W of Konya (37.88° N 32.34° E), 06.vii.1962; A. Giordani Soika leg. (CMK).—1♀; Mardin: 15 km N Midyat (37.55° N 41.36° E); 22.vi.1997; Ma. Halada leg. (CMK).—1♀; Nevşehir: Ürgüp (38.63° N 34.90° E), 1070 m; 26.–29.vi.1977; J. Heinrich leg. (CMK).—2♀; Urfa (37.17° N 38.78° E), 530 m; 1.vi.1968 and 28.v.1970; J. Gusenleitner leg. (CMK).—2♂; Urfa: Halfeti (37.24° N 37.86° E), 427 m; 28.v.1978; M. Schwarz leg. (CMK).—1♀; Urfa: S of Harran (36.86° N 39.02° E); 02.vi.1977; K. Warncke leg. (CMK).

*Material not examined*. GREECE: Attica, Dionsos [=Dionysos] (38.09° N 23.87° E) [9].—ISRAEL: Sede Boquer, 51 km S Beer Sheva (type locality of *S. bicornuta*) (38.87° N 34.79° E) [11].—IRAN: Fars, 20 km W of Neyriz, 1550 m; K. Warncke leg. [44].—TURKEY: Urfa, Birecik (37.02° N 37.97° E) [13].—Erzurum: Olur, Süngübayır (40.88° N 42.09° E) (Naturalis Biodiversity Center, RMNH.INS.1662642; BOLD).

*Diagnosis/Description*. A detailed description, supported by line drawings and photographs, is available in the literature [5].

*Genetic analysis*. The DNA sequence of a specimen from Turkey clusters with *S. surica* sp. nov. from which it has a genetic distance of 9.4–10.2% and with *S. alainensis* sp. nov. with a distance of 9.5%. The genetic distance to *S. aegyptiaca* varies between 10.5 and 11.1%, and to *S. nasuta* between 11.7 and 13.9%.

*Distribution*. The distribution extends from, Greece over Turkey to southern Iran and includes the Levantine. Countries: Greece, Iran, Israel, Palestine, Syria, and Turkey (Figure 8).

**Figure 17:**
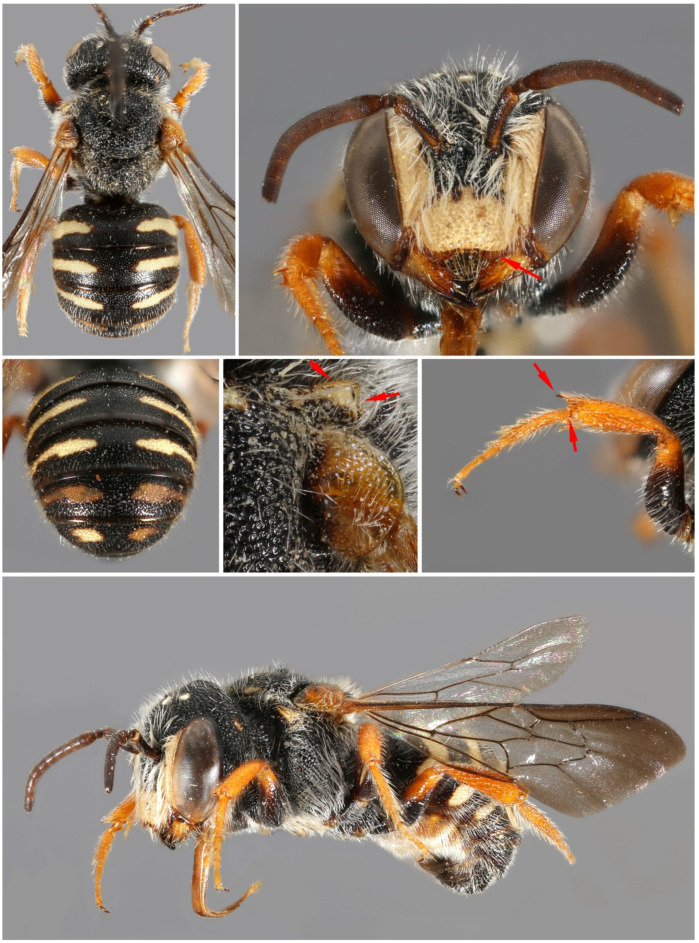
*Stelis surica* Kasparek sp. nov.; male holotype from Oman.

**Figure 18:**
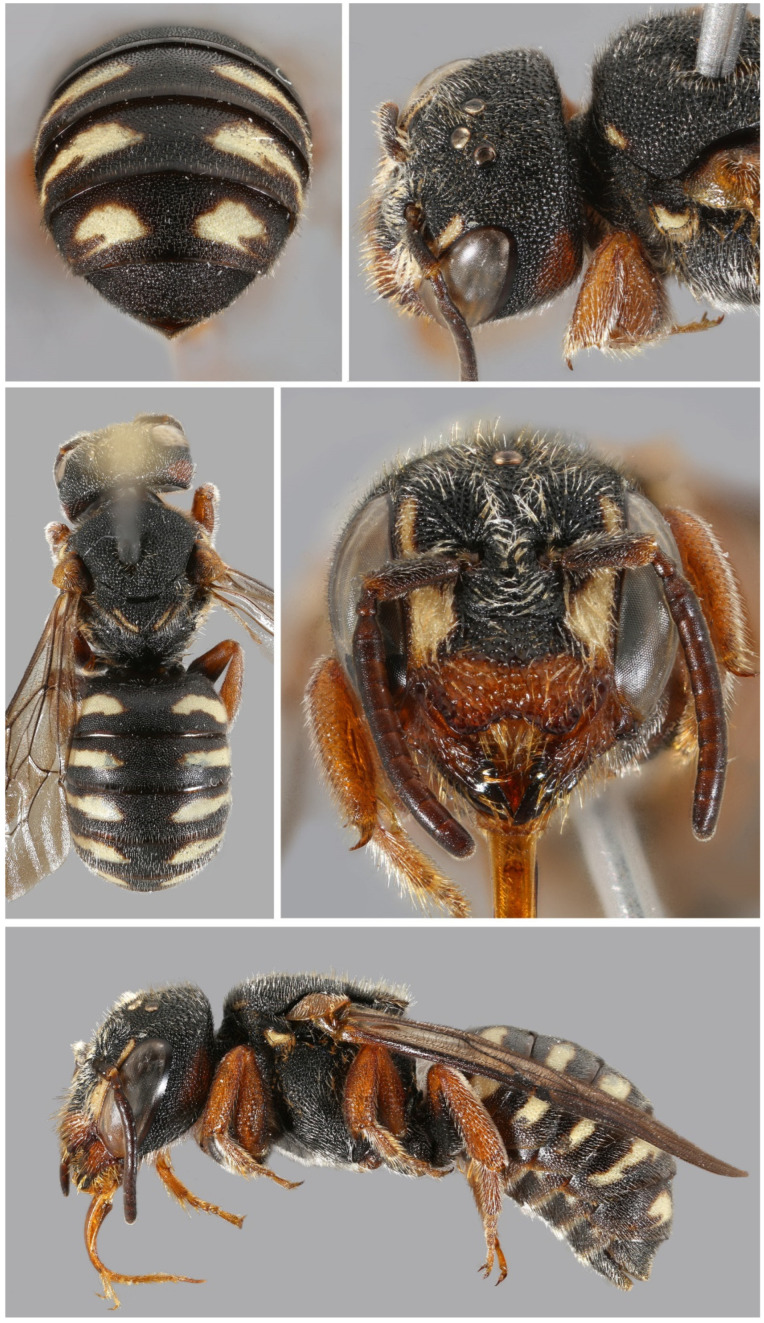
*Stelis surica* Kasparek sp. nov.; female paratype from the United Arab Emirates.

***Stelis surica* Kasparek sp. nov.**
  (Figure 17 and Figure 18)

*Material examined*. HOLOTYPE: Male. Oman: SW of Sur (22.52° N 59.46° E), 03.–05.iii.2017, M. Snizek leg. (coll. Max Kasparek).—1♂, UAE: Wadi Maidaq (25.18° N 56.07° E); 04.–15.ii.2006; leg. A. v. Harten (DEI: Hym-01067); listed in [36] as *S. aegyptiaca*.—1♀; UAE: Wadi Helo (24.97° N 56.22° E); 11.–19.iii.2009; leg. Ch. Schmid-Egger (CSE).—3♀; UAE: Jebel Jibir (25.65° N 55.11° E); 21.iv.2011; leg. T. v. Harten (CSE).

*Diagnosis*. The male is characterized by dark antennae, which it shares with *S. nasuta*. From this species, it is distinguished by a high anterior lamella of the pronotal lobe (low carina in *S. nasuta*). Additionally, the clypeal punctation is coarser in *S. surica* sp. nov. than in *S. nasuta*. The female of *S. surica* sp. nov. is characterized by a reddish-brown clypeus with broad black base and black apical rim. *Stelis nasuta* and *S. aegyptiaca* have black clypei, *S. alainensis* sp. nov. and *S. pentelica* yellow or whitish clypei with brown apical rim.

*Derivatio nominis*. The species is named after Sur, a coastal town to the southeast of Muscat, Oman, where the holotype was collected.

*Barcode Index Number (BIN)*: BOLD:AFS3414 (based on the holotype and three paratypes).

*Description*. *Female*. Body length: 7–8 mm; intertegular length: 1.69–2.30 mm; marginal cell length: 1.48–2.03 mm.—*Head*: Black with white paraocular area (broad in the lower paraocular area, tapering in the upper part). Clypeus coarsely punctate; base black, median part pale yellow with reddish-brown tinge; apical rim broadly brown. Red-brown preoccipital band reaching to mid-eye, interrupted medially (broad ventrally, tapering towards the middle); may be reduced to a red-brown spot or entirely absent (in one specimen). Mandible red-brown with one large apical and one very small basal tooth.—*Mesosoma*: Scutum dorsally convex, with yellow band along anterior margin (reduced to small remnants in two specimens); punctation dense and shallow. Scutellum elongate, apex slightly truncate; outer margin lamellate; yellow stripe laterally. Punctures on scutum and axilla equal in size, larger on scutellum. Pronotal lobe with anterior lamella. Legs orange-brown except for black coxae and anterior femora.—*Metasoma*: T1-T5 with large off-white bands, not reaching the middle; bands attenuated anteriorly and posteriorly. T6 black with finely erose apical margin. S6 semi-circular with slightly pointed tip.

*Male*. Body length: 7.0 mm; intertegular length 1.96 mm; marginal cell length: 1.66 mm.—*Head*: Head black with a pale yellow clypeus (except for two brown spots at the base and the apical rim), paraocular area, and a minute spot behind the eye; clypeus with thick, shiny, crystal clear, transparent cuticle; apical rim bilobed, semi-transparent light brown; mandible yellow at base, light brown medially and black at the apex (teeth); antenna greyish brown; narrow yellowish stripe on underside of scape.—*Mesosoma*: Black with a small yellow rectangular spot at anterior margin; shallow emargination at the outer border between scutellum and axillae; punctures on scutum large with small, shiny ridges in between; punctation of scutum similar, but with large punctures; pronotal lobe black and base and yellow distally; anterior and lateral edges with high lamellae, which intersect at right angles. Femora black with orange apices; tibiae and tarsi orange. Outer one third of wing strongly infuscated.—*Metasoma*: Black with pale yellow longish spots on T1 to T5; yellow maculation absent in T6 and T7; posterior margins of maculations not as a smooth line, somewhat ragged; punctation dense, interstices narrow, shiny; posterior impunctate margins of terga broad, dark brown, shiny. Apical margin of S2 with a fringe of equally long white hairs; S3 medially widely emarginate, with hairs conspicuously longer medially than laterally; black comb on S4 medially.

*Genetic analysis*. The sequences of the DNA of the COI gene were successfully retrieved from four specimens, the holotype male and three paratype females. Intraspecific genetic distance ranged from 0.2 to 0.7%. The closest matches with other species are *S. pentelica* (genetic distance 9.4–10.2%), *S. nasuta* (11.5–15.2%), *S. alainensis* sp. nov. (11.9–12.7%), and *S. aegyptiaca* (12.0–12.6%).

*Distribution*. Only known from Oman and the United Arab Emirates (Figure 8).

**Identification Key for the species of the subgenus *Stelis* (*Stelidomorpha*)**

***Females***
  1 Clypeus entirely black……………………………………………………………………………………………………………………………………………………2
  – Clypeus different.…………………………………………………………………………………………………………………………………………………………4
  2 Antenna dark grey or black..……………………………………………………………………………………………………………………………………*S. nasuta*
  – Antennal scape and underside of flagellomeres orange..……………………………………………………………………………………………………………3
  3 T1 with lateral bands not reaching the middle; tergal maculation yellow, occasionally bordered with reddish or brown tones at the edges.…………………………………………………………………………………………………………………………………………………………..*S. aegyptiaca*
  – T1 with uninterrupted tergal band; tergal maculation orange-red.……………………………………………………………………………*S. canaria* stat. nov.
  4 Clypeus dirty yellowish or orange-brown with black base and a broad black apical rim..……………………………………………………*S. surica* sp. nov.
  – Clypeus whitish or yellow with broad brown apical rim……………………………………………………………………………………………………………5
  5 Vertex short, 2.6–2.8x hind ocellus diameter……………………………………………………………………………………………………*S. alainensis* sp. nov.
  – Vertex long, 4.2–5.0x hind ocellus diameter..………………………………………………………………………………………………………………*S. pentelica*
***Males***
  1 Antenna dark (dark grey, dark brown or black)..…………………………………………………………………………………………………………………….2
  – Antenna predominantly orange or red-brown..………………………………………………………………………………………………………………………3
  2 Clypeus with fine, sometimes confluent punctures; pronotal lobe with anterior carina or low, indistinct lamella…………………………………*S. nasuta*
  – Clypeus with coarse punctation; pronotal lobe with high anterior lamella..……………………………………………………………………*S. surica* sp. nov.
  3 Scutellum and axillae in dorsal view V-shaped with emarginate apex…………………………………………………………………………………*S. pentelica*
  – Scutellum and axillae widely semicircular..…………………………………………………………………………………………………………………………..4
  4 T1 with uninterrupted orange-red band; metasomal terga with orange-red maculation..……………………………………………..…..*S. canaria* stat. nov.
  – T1 with lateral yellow or light orange bands; metasomal terga with yellow or whitish maculation..…………………………………………………………5
  5 Pale yellow lateral tergal bands on T1–T3 reaching the lateral margin at its contact with their corresponding sterna..………………*S. alainensis* sp. nov.
  – Pale yellow mediolateral bands on T1–T3 not reaching the lateral margin..……………………………………………………………………..…..*S. aegyptiaca*

## 4. Discussion

The number of species in the subgenus *Stelidomorpha* has increased from three to six with the description of two new species from the Arabian Peninsula and the elevation of a subspecies to species rank. Additionally, the discovery that the megapopulation of *S. nasuta s.l.* comprises three distinct evolutionary lineages—likely qualifying for species status—suggests that the total number of *Stelidomorpha* species may rise to eight. While the newly described species were discovered in the relatively understudied Arabian Peninsula, the new putative species were identified in well-studied Europe. This also suggests that bee diversity may be significantly higher even in well-studied regions than our classical species inventories suggest and underlines the role of genetic analyses to unveil this diversity. In a comprehensive genetic study of 503 named Central European morphospecies of wild bees with available DNA barcodes, 25 species were assigned to two or more Barcode Index Numbers (BINs) [45]. Additionally, the authors compared specimens of 191 named morphospecies with available barcodes from both Central and Southern Europe. Among these, specimens from 23 species were assigned to different BINs in the two regions. These figures support the idea that our classical species lists do not sufficiently reflect the actual diversity. The authors of this study explain multiple BINs for a single morphospecies with a high level of intraspecific variation but also consider still undiscovered cryptic species [45]. With the support of genetic barcoding, many new bee species were described in recent years, and often they were referred to as “hidden diversity” or “cryptic diversity” (e.g., [46,47,48,49,50,51,52,53]). While the term “cryptic species” usually refers to distinct species that are morphologically indistinguishable but can be differentiated using genetic markers [54,55,56], some authors use this term also in a broader sense and include morphologically similar, yet clearly distinguishable species (e.g., [48,49,56,57,58]). In such cases, terms such as “near-cryptic”, “semicryptic” or “pseudocryptic” are sometimes applied (e.g., [59,60]).

The taxonomic treatment of genetic lineages referring to the same morphospecies remains ambiguous in the literature. Based on the phylogenetic species concept [61,62] that defines species as the smallest group of individuals that share a common ancestor and possess unique, derived characteristics, the genetic lineages—lineages like those three lineages identified here in *Stelis nasuta*—could be treated as species, although no morphological differences were identified. However, the phylogenetic species concept relying on evolutionary history is often in conflict with the biological species concept that relies on reproductive isolation as the primary criterion for defining species boundaries. The question of whether genetic lineages identified by species delimitation programs should be recognized as valid species in the absence of additional evidence remains a subject of debate [63]. Controversy has emerged from cases where new species were described almost exclusively on the basis of COI barcodes—for instance, dozens of Braconidae (Hymenoptera) from Costa Rica [64] and ten Hesperiidae (Lepidoptera) from the same region [65]. These practices have been strongly criticized [66], and the authors of the latter study even acknowledged that their species descriptions were based solely on DNA sequences without examining specimens. In contrast, a study of 242 European leaf-mining moths of the family Gracillariidae, which revealed 13 morphospecies corresponding to 27 BINs, applied a more differentiated approach [67]. The authors classified these clusters—where genetic divergence was detected but no morphological or ecological differences were found—as “deep conspecific lineages” (DCL). DCLs refer to individuals of a currently accepted species that fall into multiple BINs but for which detailed morphological and ecological analyses do not justify recognition as separate species [67].

A literature review found that fewer than 30% of the studies reviewed made taxonomic recommendations and only 25% describe new species [68]. This raised the question of whether the authors lacked confidence in their own species delimitation analyses, were unable to reconcile conflicting methods, or tacitly acknowledged the insufficiency of their data. Other authors have assigned codes to certain haplotype clusters [50,65] without providing formal names and species descriptions. Moreover, the description of morphologically indistinguishable species based solely on genetic data would pose a major challenge, particularly in relation to compliance with the International Code of Zoological Nomenclature (ICZN) (e.g., [53]).

The widespread scepticism toward the results of genetic species delimitation studies has supported the rise in integrative taxonomy which combines multiple lines of evidence (e.g., morphology, genetics, ecology, behaviour) in species delimitation and description. We interpret the principle of taxonomy by congruence [69] to mean that the delimitation of a species based on a single genetic marker must be supported by at least one additional independent genetic marker or by morphological or biological evidence in order to be considered for formal species recognition (see also, e.g., [70]).

Limited studies have been carried out in wild bees. For anthidiine bees, a study of COI sequences of *Anthidium dalmaticum* Mocsáry, 1884 revealed three distinct OTUs, with each of them qualifying as a distinct species [71]. However, since no morphological or morphometric characters supported the delimitation of these groups, they were regarded as cryptic candidate species, and all of them were assigned to *A. dalmaticum* until additional evidence for their distinctiveness becomes available. Similarly, three distinct lineages were found for *Anthidium undulatum* Dours, 1863 [72]. While no morphological traits were fully diagnostic for these three groups, they were clearly distinguished by morphometric traits. Remarkably, two of the three OTUs were found to coexist within the same geographic area and the same habitat. Consequently, all three OTUs were formally described as distinct species.

Also, in *Stelis nasuta*, all three OTUs qualified as distinct species according to the algorithms of all four species partition programs (ABGD, ASAP, bPTP, and RESL). However, the justification for species assignment is based solely on a single gene and lacks support from other morphological or biological characteristics. Therefore, in line with common practice, we refrain from formally describing them as new species and assigning names under ICZN standards. Instead, we consider these three OTUs as candidate species.

In *S. nasuta*, the ASAP program for automatic species partitioning suggested as many as 14–16 partitions (=“putative species”) as the best options. However, only a further analysis of the partition composition and the geographic distribution of their members allowed for the reduction in this number to three, indicating that ASAP tended to oversplit the gene sequences. By contrast, the ABGD program yielded three partitions from the beginning, but returned five partitions in a recursive analysis. These additional partitions were discarded as they did not align with the observed geographic distribution pattern. The bPTP analysis also identified three partitions, though it suggested the possibility of finer subdivisions. Finally, RESL (BOLD) returned three partitions and assigned distinct Barcode Index Numbers (BINs) to them. While all four approaches identified the same three partitions as putative species, the varying processes and results highlight the challenges and potential pitfalls involved in species delimitation.

Similar cases have also been reported in other bees, for example, in *Thyreus truncatus* (Pérez, 1884), which exhibits three clearly distinct genetic clades [73]. However, unlike *S. nasuta*, these clades occur further west (in Iberia, southern France, and extending eastwards from eastern France). In the absence of reliable morphological characters, the authors refrained from elevating these genetic lineages to species rank.

It is well known that different species delimitation methods can yield varying results, and oversplitting is much more common than overlumping. For instance, a study on Asian coleopterans found that bPTP tends to oversplit [74], and similar findings were reported for Sri Lankan coleopterans, where most species delimitation methods, including bPTP, ABGD, ASAP, and RESL, resulted in oversplitting [75]. Possible causes of oversplitting include singletons (OTUs represented by a single sequence) or the absence of intermediate haplotypes due to large unsampled geographic areas [75,76,77]. To mitigate these challenges and obtain robust results, it was recommended to apply multiple methods in parallel and rely on the results only when they are congruent [78]. Following this approach, we performed our analysis of *Stelis nasuta* with four different programs and concluded that genetic evidence supports the idea that the name *S. nasuta* apparently refers to three distinct species, which, however, await further confirmation through at least one additional line of further evidence (dual evidence approach) before names can be assigned.

Our results indicate that the members of the three lineages of *S. nasuta* live in the same wider area, but in geographically well-separated parts with no overlap. Interbreeding between these lineages is not known and the genetic analysis suggests that there is strict isolation. The three lineages exhibit patterns consistent with parapatric species, yet the factors underlying this disrupted gene flow remain unclear. It is possible that the morphometric traits examined did not capture those that distinguish the groups. Analyses incorporating additional morphological parameters or employing more sophisticated morphometric approaches [79] may reveal subtle differences among these lineages. Beyond morphology, potential behavioural or ecological distinctions should also be considered. As *Stelis nasuta* is a cleptoparasite with a high level of host-specificity, attention may be directed toward host–parasite relationships. While *Megachile parietina* (Geoffroy, 1785) [=*Chalicodoma muraria* Latreille] is the generally known host of *S. nasuta* in Central Europe [80,81], *M. pyrenaica* Lepeletier, 1841 and *M. sicula* (Rossi, 1792) have been reported from southern Europe [5,82]. It would be valuable to investigate whether there exist regional differences in host selection and, if so, whether such differences can be linked to the different lineages of *S. nasuta*.

## Figures and Tables

**Table 1:** Specimen data of *Stelis nasuta* used for the genetic study. Region: Central, SW, and SE refer to the three lineages identified in this study; BIN = Barcode Index Number of the BOLD database; bp = number of base pairs.

Region	BIN	BOLD Specimen Page	Country	Year	Collector	bp
Central	BOLD:AAO3679	BC ZSM HYM 02269	Italy	1995	C. Schmid-Egger	426
Central	BOLD:AAO3679	BC ZSM HYM 02270	Italy	1995	C. Schmid-Egger	620
Central	BOLD:AAO3679	BC ZSM HYM 02271	Italy	1995	C. Schmid-Egger	620
Central	BOLD:AAO3679	BC ZSM HYM 02272	Italy	1995	C. Schmid-Egger	425
Central	BOLD:AAO3679	BC ZSM HYM 05944	Croatia	2004	E. Scheuchl	657
Central	BOLD:AAO3679	BC ZSM HYM 07065	France	2010	C. Schmid-Egger	658
Central	BOLD:AAO3679	BC ZSM HYM 07066	France	2010	C. Schmid-Egger	658
Central	BOLD:AAO3679	BC ZSM HYM 07067	France	2010	C. Schmid-Egger	658
Central	BOLD:AAO3679	BC ZSM HYM 07068	France	2010	C. Schmid-Egger	631
Central	BOLD:AAO3679	PAR_167	Italy	2019	F. R. Dani	658
Central	BOLD:AAO3679	PAR_168	Italy	2019	F. R. Dani	658
Central	BOLD:AAO3679	UNIFI1269-24	Italy	2024	M. Bonifacino	658
Central	BOLD:AAO3679	MK-seg421	Greece	2016	C. Schmid-Egger	658
SW	BOLD:AEC4732	BC-LPRCorse1976	France: Corsica	2019	R. Le Divelec	653
SW	BOLD:AEC4732	BC-LPRCorse1977	France: Corsica	2019	R. Le Divelec	653
SW	BOLD:AEC4732	INV12000	Spain	2021	T. J. Wood	659
SW	BOLD:AEC4732	MK-tjw226	Spain	2021	T. J. Wood	627
SW	BOLD:AEC4732	MK-tjw335	Spain	2022	T. J. Wood	632
SW	BOLD:AEC4732	MK-nou153	Algeria	2023	N. Benarfa	569
SW	BOLD:AEC4732	MK-nou190	Algeria	2023	N. Benarfa	610
SW	BOLD:AEC4732	MK-mons728	Morocco	2023	A. Sentil	633
SW	BOLD:AEC4732	GIU43_APCUL	Italy (Sardinia)	2023	M. Annessi/A. Di Giulio	619
SE	BOLD:ABW1388	MK-whl007	Jordan	2016	J. Gebert	599
SE	BOLD:ABW1388	MK-oll0848	Israel	2019	M. Halada	631
SE	BOLD:ABW1388	MK-mk1237	Bulgaria	2022	Z. Haladova	634
SE	BOLD:ABW1388	MK-tuz041	Lebanon	2023	V. Soon	658
SE	BOLD:ABW1388	MK-mk1654	Turkey	2024	M. Kasparek/O. Özgül	627

## Data Availability

Genetic data were uploaded to the BOLD database https://www.boldsystems.org and will be made publicly available after publication of this paper. The relevant BINs are given here in the species accounts, for *S. nasuta* in Figure 11. All distribution data of *Stelis nasuta* are included in the Supplementary File.

## Abbreviations

The following abbreviations are used in this manuscript:

CMK	Collection Max Kasparek, Heidelberg (Germany)
CSE	Collection Christian Schmid-Egger, Berlin (Germany)
DEI	Senckenberg Deutsches Entomologisches Institut, Müncheberg (Germany)
